# Beyond associations: methodological and conceptual challenges in diet–microbiome research for cardiometabolic risk

**DOI:** 10.3389/fnut.2026.1913324

**Published:** 2026-10-13

**Authors:** Merve Çapaş, Efsane Yavuz Bedir, Kübra Ergan, Beyza Arı Gedik, Beraat Dener, Duygu Agagündüz

**Affiliations:** 1 Department of Nutrition and Dietetics, Faculty of Health Sciences, Erciyes University, Kayseri, Türkiye; 2 Department of Medical Services and Techniques, Vocational School of Health Services, Giresun University, Giresun, Türkiye; 3 Department of Nutrition and Dietetics, Faculty of Health Sciences, Istanbul Yeni Yüzyil University, Istanbul, Türkiye; 4 Department of Nutrition and Dietetics, Faculty of Health Sciences, Selcuk University, Konya, Türkiye; 5 Department of Nutrition and Dietetics, Faculty of Health Sciences, Bingöl University, Bingöl, Türkiye; 6 Department of Nutrition and Dietetics, Faculty of Health Sciences, Gazi University, Ankara, Türkiye

**Keywords:** bioactive nutrients, cardiometabolic health, diet, gut microbiome, multi-omics, personalized nutrition

## 1 Introduction

The multifaceted nature of the interactions between diet, microbiota, and host plays a critical role in the pathogenesis of cardiometabolic diseases. Dietary patterns directly modulate the composition and metabolic outputs of the gut microbiota, shaping the cardiovascular risk profile through pathways such as bile acid production, short-chain fatty acid synthesis, and trimethylamine N-oxide (TMAO) metabolism (1). These diet-derived microbial pathways provide a mechanistic basis for linking habitual dietary exposure with metabolic inflammation, vascular dysfunction, and cardiometabolic risk. However, while the relationship between circulating levels of microbial metabolites and cardiovascular phenotypes is not yet fully elucidated, the causal roles of these metabolites in pathogenesis remain unclear (2). In this context, modulation of microbial metabolism through diet holds the potential for an innovative strategy for cardiometabolic protection (3). Nevertheless, translating this potential into clinical or public health recommendations requires a clearer understanding of how dietary exposures, microbial functions, and host metabolic responses interact across heterogeneous populations. The regulatory effects of microbial metabolites on deoxyribonucleic acid (DNA) methylation and histone modifications influence cellular mechanisms involved in the pathogenesis of cardiometabolic diseases, leading to a better understanding of the multifaceted interactions between nutrition, epigenetic regulation, and host genetics (80). This article aims to critically examine the methodological, conceptual, and translational challenges in diet–microbiome–cardiometabolic research and to highlight future directions for generating more robust evidence on cardiometabolic risk.

## 2 The need for a conceptual framework on the diet-microbiome-cardiometabolic axis

Nutrition is one of the most important factors influencing gut microbiome composition. Dietary preferences such as vegetarian, vegan, low-carbohydrate, or gluten-free diets can lead to significant variations in the gut microbiota (4), and specific microbes may regulate nutrient availability to the host (5). Consequently, dietary patterns have attracted considerable attention because of their potential to influence cardiometabolic health through modulation of the gut microbiome. The gut microbiome represents a complex ecosystem encompassing microbial communities, their genetic material, and bioactive metabolites that may interact with host metabolic and cardiovascular pathways (6, 7). Cardiometabolic diseases are associated with alterations in microbiome structure and function, and diet plays an important role in this relationship. Interactions between the gut and cardiovascular system through microbial metabolites, inflammatory pathways, and neurohormonal signaling constitute the gut–heart axis (8), through which the gut microbiota may regulate lipid metabolism, inflammation, and vascular function (9). The modifiable nature of both the microbiome and nutrition offers opportunities for novel therapeutic strategies, while meta-omic approaches continue to improve our understanding of these complex interactions (10). Accordingly, the relationship between diet, the gut microbiome, and cardiometabolic health is increasingly recognized as multidimensional and highly complex (11). Likewise, beneficial effects of low-calorie diets, exercise, probiotics, and the Mediterranean diet on cardiovascular risk have been reported (12). However, despite growing evidence supporting the role of diet–microbiome interactions in cardiometabolic health, demonstrating consistent microbiome effects across large populations remains challenging (13). These inconsistencies are largely attributable to the complexity of dietary patterns, difficulties in distinguishing dietary influences from other lifestyle factors (14, 15), and substantial interindividual variability in gut microbiome composition (16).

## 3 Current epidemiological evidence in the context of cardiometabolic risk: consistency and limitations

Dietary intake and environmental factors play important roles in shaping the gut microbiota from early life (17). Although the adult gut microbiota is relatively stable, dietary composition substantially affects its structure and function (18). In particular, the A large-scale prospective cohort study of 72,113 women, using food frequency questionnaires (FFQ), demonstrated that a Western dietary intake pattern—characterized by high consumption of red meat, processed meat, refined grains, French fries, and sweets/desserts—was associated with an increased risk of total mortality, including cardiovascular disease (19), whereas plant-based dietary patterns, including high-fiber and vegetarian diets, have been associated with beneficial microbiota modulation and improvements in insulin resistance (20, 21). In the 3-month interim analysis of the first 772 participants from the large-scale cohort study PREDIMED, the Mediterranean Diet intervention was shown to favorably affect classical and emerging intermediate markers of cardiovascular risk—particularly blood pressure, insulin sensitivity, lipid profile, and circulating inflammatory markers—compared with the control diet, demonstrating the impact of dietary pattern on cardiovascular disease (81). Gut microbiota-derived metabolites have also been linked to cardiovascular disease characteristics. Trimethylamine N oxide, produced through the metabolism of dietary phosphatidylcholine by the gut microbiota, has been shown to increase macrophage foam-cell formation and the development of atherosclerotic lesions (82). This gut microbiota-derived metabolite, produced from dietary choline, has been shown—in both animal models and human subjects—to be associated with cardiovascular disease by increasing platelet hyperreactivity and thrombosis risk (22, 23). However, the reliable measurement of such microbiota-derived metabolites as biomarkers also entails significant methodological challenges related to the biological matrix used (plasma, urine, or feces). Indeed, a recent study demonstrated that the same intervention led to significant microbial metabolic changes in feces, whereas systemic levels in plasma and urine remained stable, indicating that the effect of an intervention may be overlooked when only a single matrix is evaluated (83). A randomized controlled trial conducted in patients with type 2 diabetes observed that diastolic blood pressure decreased by approximately 7 mmHg from baseline in the high-fiber diet intervention group, whereas no significant change occurred in the control group (24), similarly a systematic review suggested that the Mediterranean diet may positively influence blood pressure, insulin resistance, and inflammatory markers, although greater methodological standardization is still needed (25).

## 4 Methodological and measurement-based limitations in diet–microbiome research

The interpretation of diet–microbiome studies is challenged not only by biological complexity but also by heterogeneity in research design and measurement methods. Despite the established role of diet in shaping the gut microbial ecosystem, there is still no standardized framework for capturing dietary exposure, characterizing microbial profiles, and linking these datasets analytically (26, 27).

Frequently used dietary assessment approaches, such as FFQs, 24-h recalls, and food records, differ in their ability to characterize dietary exposure relevant to diet–microbiome associations (28). In a prospective cohort of 3,096 adults, long-term plant-based dietary patterns assessed using repeated FFQs explained more variation in gut microbial structure than short-term dietary patterns assessed using 3-day 24-hr recalls, although both were associated with microbial composition (29). Thus, exposure window and assessment method may influence observed diet–microbiome associations.

Methodological variability is also evident in microbiome analysis. Differences in sequencing methods, sample storage, DNA extraction, and bioinformatic pipelines may produce divergent findings from the same biological sample (27, 30). In a randomized crossover feeding trial, contrasting dietary patterns produced modest species-level microbial responses but altered 105 microbial functional pathways, integrated multi-omic features were also associated with cholesterol, blood pressure, and triglyceride responses (31). Thus, apparent dietary responses may depend on the analytical layer examined.

Another challenge involves integrating dietary and microbiome data. Temporal mismatches, multiple comparisons, and interindividual variability complicate the identification of meaningful biological signals (26). In 1,368 individuals, variation in the plasma metabolome was attributable to diet, gut microbiome, and genetics to different degrees across metabolites (32), highlighting the difficulty of attributing circulating metabolites to a single source.

The biological matrix introduces an additional source of methodological heterogeneity. Across three nutrition or exercise intervention datasets, fewer than 1% of significant fecal–plasma metabolite pairs were shared across all studies, indicating substantial study-specific and interindividual variability (33). These findings warrant caution when extrapolating fecal metabolite measurements to circulating exposure and host health. Moreover, integration of habitual dietary data, metagenomics, and fecal metabolomics in 2,647 individuals showed that fecal metabolites captured reported food-group intake and dietary patterns and were informative for atherosclerotic cardiovascular disease risk (34). Such molecular readouts may complement self-reported dietary assessment. Future studies should prioritize standardized protocols, microbiota-oriented dietary assessment tools, and robust integrative analytical approaches.

## 5 Heterogeneity, confounding factors, and the problem of causality

The interpretation of the diet–microbiome–cardiometabolic axis is complicated by pronounced interindividual variation in the gut microbial community. The composition of the intestinal microbiota is influenced by factors such as birth mode, demographic characteristics, medication exposure, and environmental influences, with diet acting as a key determinant (35, 36). Diet–microbiota relationships vary across studies, with environmental factors including dietary habits, migration, and lifestyle influencing microbiome composition differently by geographic origin (37–39). Dietary changes may alter the species-level microbial composition and subsequently affect host health (40), though studies are often limited by small sample sizes and insufficient dietary specificity (41). Restrictive diets are associated with elevated Bacteroides abundance (42, 43), while Western-style diets showed similar findings in earlier studies (44, 45). with interindividual variability and confounding factors complicating causal interpretation (46). Although numerous microbial–health associations have been identified (47), elucidating the underlying mechanisms remains challenging (48), and the high-dimensional complexity of microbiome data further complicates the identification of disease-associated communities (49, 50). Additionally, various pharmacological agents, including antimicrobials and cardiovascular medications, may alter microbial composition and drug response, representing a major source of confounding (51–56).

## 6 The gap between mechanistic insights and epidemiological evidence: challenges in metabolite functional interpretation

The effects of the human microbiota on cardiovascular and metabolic diseases are increasingly understood through microbial metabolites, including short-chain fatty acids, trimethylamine-N-oxide, and secondary bile acids, which regulate host physiology and cardiometabolic health (57, 58). Elucidating the biosynthesis pathways and host-body interaction mechanisms of these metabolites is of great importance for understanding disease pathogenesis and developing new therapeutic strategies (59). Despite strong mechanistic findings demonstrated in preclinical studies, the inability to consistently validate these metabolites as cardiovascular risk markers in human cohorts deepens the knowledge gap in this area (58, 60). A meta-analysis encompassing 11 prospective cohort studies showed that higher circulating TMAO levels were associated with a 23% increased risk of cardiovascular events and a 55% increased risk of all-cause mortality (61). In a study including data from nine cohort studies (10,301 patients), combined data showed that high plasma TMAO concentrations could increase the risk of major cardiovascular events by 58% in patients with coronary heart disease (62). Another study, a systematic review of 44 prospective cohort studies and 84,771 participants, associated high serum TMAO levels with an increased risk of total cardiovascular disease, cardiovascular mortality, and all-cause mortality (63). When examining clinical intervention studies, findings of the same frequency and certainty as those observed in epidemiological studies have not been found (64).

Similarly, while inhibition of TMA production or reduction of TMAO levels in animal models may limit atherosclerosis and platelet activation, the failure of dietary changes and probiotic interventions to consistently reduce TMAO in human studies prevents the direct translation of mechanistic findings to clinical treatment response. Although the evidence regarding TMAO is mixed, it is considered a promising future treatment approach for atherosclerosis (65). Therefore, the key gap in the clinical use of TMAO is not the prognostic value of a single baseline measure, but rather the need for serial measurements combined with diet, renal function, medication use, and microbial production capacity, and for randomized trials to demonstrate whether interventions that reduce TMAO actually improve cardiovascular outcomes.

This inconsistency stems not only from microbial composition but also from the complexity of interactions between specific metabolites and the host, and from the diversity of environmental and genetic factors that modulate these interactions (66). Elucidating the dynamic interactions between microbial metabolomic profile and host physiology is critical to resolving these inconsistencies in the pathogenesis of cardiometabolic diseases (66, 67). Although the systemic effects of microbiome-derived bioactive compounds on obesity, insulin resistance, and atherosclerosis remain unclear, gene-scale metabolic models and advanced molecular analyses may provide important mechanistic insights into interindividual treatment responses (2, 68).

## 7 Future perspectives: system approaches, multi-omics, and the transition toward personalized nutrition

The complexity of diet–microbiota–host interactions, individual-level confounding factors, and baseline microbiota composition complicate the standardization of personalized nutrition strategies. In addition, phenotypic characteristics such as age, sex, and disease status should be considered, while shotgun metagenomic/WGS approaches may provide more comprehensive functional assessments compared with 16S rRNA analyses (69). Furthermore, reliable identification of microbiome-based cardiometabolic biomarkers requires dense longitudinal sampling, comprehensive metadata, and large cohort studies (10).

Despite these advances, difficulties in dietary assessment, the lack of validated biomarkers, inadequacies in food composition databases, and standardization problems in sample collection and bioinformatic workflows remain major methodological limitations in nutrition–microbiota research. Therefore, multidisciplinary studies supported by adequate sample sizes and effective integration of meta-omics data are expected to substantially contribute to future progress in the field (70, 84). Overall, personalized nutrition approaches appear promising for the management of cardiometabolic disease, however, standardized methodologies and large-scale, long-term studies are still required before routine clinical implementation (Figure 1).

**Figure 1:**
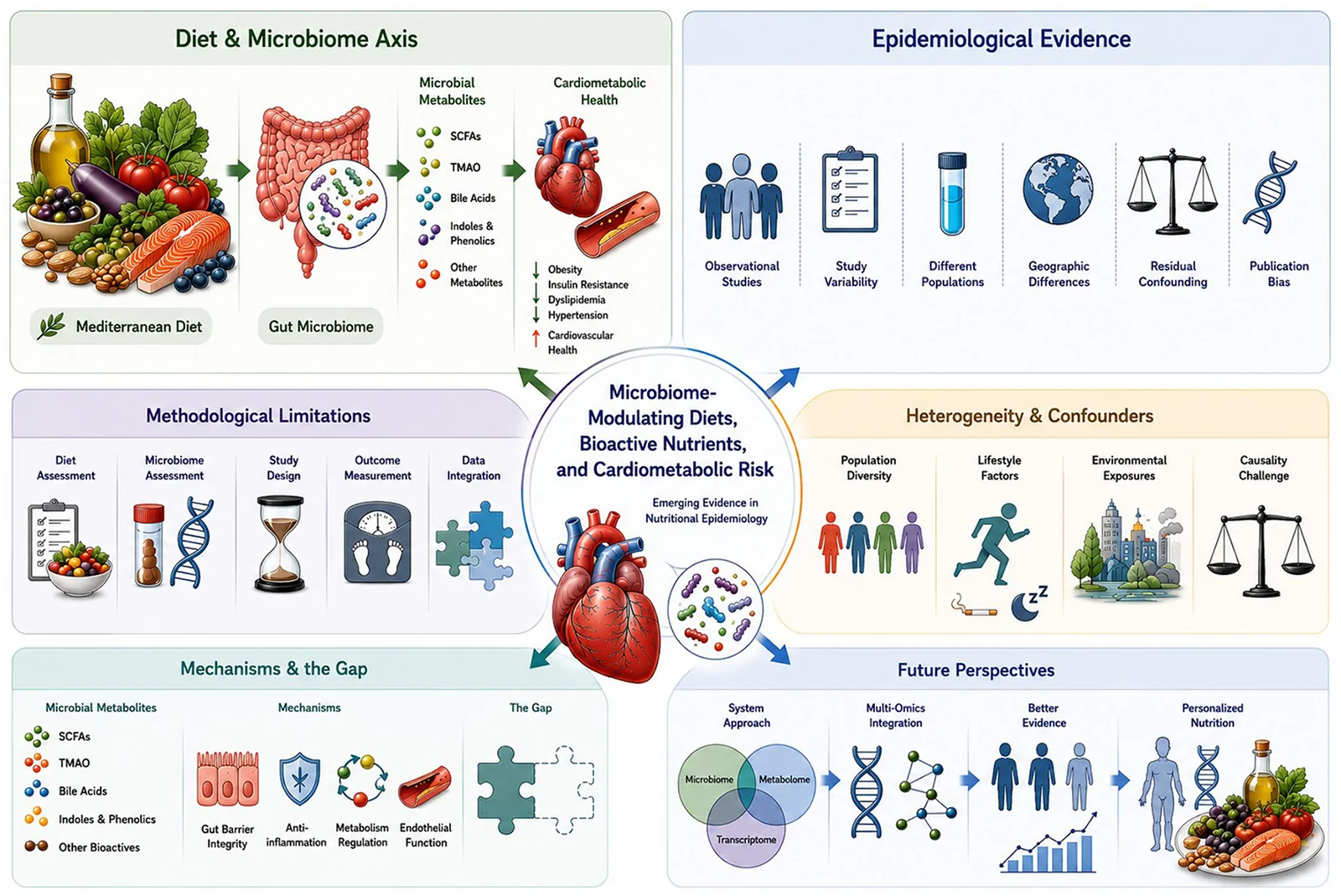
Diet–gut microbiome–cardiometabolic axis: evidence, methodological challenges, mechanistic gaps, and future perspectives.

## 8 Discussion

The diet–microbiota–cardiometabolic axis stands out as one of the most complex, yet rapidly developing, research areas in modern nutritional epidemiology, offering significant scientific opportunities. A key strength of the current literature is that research is no longer limited to characterizing the composition of the gut microbiota, it is now beginning to evaluate dietary components, microbial functions, microbiota-derived metabolites, and host cardiometabolic responses within the same biological framework. Importantly, the integration of these complementary approaches provides an opportunity to move beyond simple associations and investigate the biological pathway linking dietary exposure, microbial activity, circulating metabolites, and cardiometabolic outcomes (71). However, the central challenge is now to determine which of these relationships are reproducible, causal, and clinically meaningful.

The heterogeneity of findings in the literature, particularly the discrepancies between fecal and circulating SCFA levels and the conflicting effects of these metabolites on host receptors, prevents a complete explanation (72). Therefore, the fundamental need of this field is not only to identify new microbiota-health relationships, but also to determine under what conditions these relationships are biologically meaningful, replicable, and causal (73).

Trimethylamine N-oxide (TMAO) and short-chain fatty acids are two important examples that together reveal the strengths and weaknesses of the current evidence in this field. Although epidemiological evidence consistently links circulating TMAO with cardiovascular risk, the less consistent response observed in intervention studies highlights the distinction between prognostic association and causal modifiability (22). A similar challenge applies to SCFAs. Acetate, propionate, and butyrate are important mechanistic candidates because they are produced through dietary fiber fermentation and have been associated with blood pressure regulation, insulin sensitivity, and vascular function. However, discrepancies between fecal and circulating SCFA concentrations, together with differences in receptor-mediated effects, complicate the interpretation of their cardiometabolic relevance (74). Together, these examples underscore a major unresolved question: whether microbiota-derived metabolites primarily represent biomarkers of cardiometabolic risk, causal mediators, or potentially modifiable therapeutic targets.

The methodological variability observed in diet-microbiota research poses a significant limitation in reliably interpreting findings and comparing the results of different studies. Traditional diet assessment methods may not adequately capture dietary components, particularly those related to microbial metabolism (75), and differences in sequencing platforms, analytical processes, and bioinformatics operations further limit inter-study comparability (76).

The issue of causality remains one of the most fundamental problems in this field. Most observational studies fail to answer whether changes in the microbiota cause disease or whether disease shapes the microbiota (77). The increasing experimental evidence suggesting that metabolites such as SCFA, TMAO, and secondary bile acids may affect host physiology and cardiometabolic health highlights the importance of evaluating not only microbial composition but also microbial function (78).

Consequently, it is clear that uniform dietary recommendations do not produce similar microbial and metabolic responses in every individual. This shift will facilitate the development of more sustainable and effective clinical strategies for the prevention and management of cardiometabolic diseases. Inter-individual microbial, genetic, and metabolic variability presents both a significant methodological challenge and a major opportunity for developing personalized nutrition approaches. The fact that different individuals exhibit different microbial and metabolic responses to the same dietary intervention suggests that uniform nutritional recommendations may not produce the same biological outcome in every individual. Therefore, it is recommended that future strategies focus on multi-omics and AI-powered precision nutrition approaches that take into account an individual's baseline microbiota profile, metabolic phenotype, and lifestyle. However, since a significant portion of the current evidence is based on short-term interventions and small sample sizes, large-scale and long-term randomized controlled trials are needed to translate these approaches into clinical practice (79). Ultimately, progress in this field will depend less on identifying additional associations than on establishing which diet–microbiome relationships are reproducible, causal, and sufficiently robust to inform clinical practice.
